# Selection of Atmospheric Environmental Monitoring Sites based on Geographic Parameters Extraction of GIS and Fuzzy Matter-Element Analysis

**DOI:** 10.1371/journal.pone.0123766

**Published:** 2015-04-29

**Authors:** Jianfa Wu, Dahao Peng, Jianhao Ma, Li Zhao, Ce Sun, Huanzhang Ling

**Affiliations:** 1 College of Automation, Harbin Engineering University, Harbin, Heilongjiang, China; 2 College of Computer Science and Technology, Harbin Engineering University, Harbin, Heilongjiang, China; 3 College of National Secrecy, Harbin Engineering University, Harbin, Heilongjiang, China; 4 College of Materials Science and Chemical Engineering, Harbin Engineering University, Harbin, Heilongjiang, China; 5 College of Science, Harbin Engineering University, Harbin, Heilongjiang, China; Tsinghua University, CHINA

## Abstract

To effectively monitor the atmospheric quality of small-scale areas, it is necessary to optimize the locations of the monitoring sites. This study combined geographic parameters extraction by GIS with fuzzy matter-element analysis. Geographic coordinates were extracted by GIS and transformed into rectangular coordinates. These coordinates were input into the Gaussian plume model to calculate the pollutant concentration at each site. Fuzzy matter-element analysis, which is used to solve incompatible problems, was used to select the locations of sites. The matter element matrices were established according to the concentration parameters. The comprehensive correlation functions *K_A_ (x_j_)* and *K_B_ (x_j_)*, which reflect the degree of correlation among monitoring indices, were solved for each site, and a scatter diagram of the sites was drawn to determine the final positions of the sites based on the functions. The sites could be classified and ultimately selected by the scatter diagram. An actual case was tested, and the results showed that 5 positions can be used for monitoring, and the locations conformed to the technical standard. In the results of this paper, the hierarchical clustering method was used to improve the methods. The sites were classified into 5 types, and 7 locations were selected. Five of the 7 locations were completely identical to the sites determined by fuzzy matter-element analysis. The selections according to these two methods are similar, and these methods can be used in combination. In contrast to traditional methods, this study monitors the isolated point pollutant source within a small range, which can reduce the cost of monitoring.

## Introduction

As a result of rapid economic development, environmental pollution has become increasingly serious, and thus, it is essential to effectively monitor the atmospheric environment. The potential risks of pollution can be decreased by effectively establishing monitoring sites. Such monitoring sites consist of automatic air quality monitoring stations and have building costs that can reach several million RMB. Because of the limited funds available for environmental monitoring, the number of monitoring sites should be reduced as much as possible while ensuring the representativeness of monitoring indices, thereby improving monitoring efficiency.

Traditional methods for selecting atmospheric environmental monitoring sites (including water quality monitoring) were based on using pre-existing monitoring sites to measure the Pollutant Standards Index (PSI) followed by mathematic methods, such as fuzzy matter-element analysis and fuzzy cluster analysis, to analyze each PSI and refine the selection of suitable sites [1–6]. Alternatively, the mesh point method [7, 8] was used to divide the monitoring area into uniform-mesh grids, and each PSI was measured by establishing a sampling point in the center of each grid space, and then the locations of sites were optimized using mathematic methods. However, these methods have limitations. The traditional methods are based on pre-existing monitoring sites whose locations may not be optimal because of historical reasons and other factors. The mesh point method requires checking every measurement, which is expensive. These methods are only appropriate for monitoring air quality over a large area with several sources of pollution, such as cities, and are not suitable for monitoring the impact of a single source of pollution over a partial area, such as the impact of a factory on the nearby area. With the development of GIS and computer simulation technology, it is possible to accurately record the spread of atmospheric contamination in different types of terrain. The geographic parameters (elevation and latitude-longitude) in a certain area can be extracted by GIS, and the concentrations of pollutants can be calculated through diffusion equations using digital computation in MATLAB.

International research on atmospheric environmental monitoring includes using GIS alone to study the diffusion of atmospheric pollutants [9–13] and using fuzzy matter-element analysis to optimize pre-existing monitoring sites [14]. However, research on the combination of these methods remains sparse. Combining the two methods, to not only simulate the spread of pollutants accurately with GIS but also take advantage of the merits of fuzzy matter-element analysis, which can solve incompatible problems in environmental monitoring [15, 16], enables site selection that is not restricted by pre-existing monitoring sites. As such, site locations can be further optimized, leading to a reduction in the cost of monitoring and the efficient allocation of limited resources.

## Materials and Methods

### Gaussian plume model

The classical Gaussian plume is a steady-state model that requires a continuous release of a contaminant. The overall average plume shape is approximated by time averages sufficient to smooth the effects of plume meandering. The equation for the Gaussian plume is a function of the mean wind speed (assumed constant) and the crosswind and vertical standard deviations (*σ*_*y*_ and *σ*_*z*_). The source strength, *Q*, is the mass of material released per unit time. The time-averaged wind speed, *v*, is uniform. The contaminant concentration, *C*(*x*,*y*,*z*), is given by the following:

$$C(x,y,z,H)=\frac{Q}{2\pi \sigma_{y}^{2}\sigma_{z}^{2}v}\exp(-\frac{y^{2}}{2\sigma_{y}})\{\exp[-\frac{{\left(z-H\right)}^{2}}{2\sigma_{y}^{2}}]+\exp[-\frac{{\left(z+H\right)}^{2}}{2\sigma_{z}^{2}}]\}$$ (1)

where *σ*_*y*_ is the standard deviation of *C*(*x*,*y*,*z*) in the cross-wind direction, *σ*_*z*_ is the standard deviation of *C* in the vertical direction and *H* is the equivalent height. These dispersion parameters are functions of only the downwind direction, *x*. The *z*-dependent terms model the trapping effect of the ground by simulating a mirror source at distance *H* below the ground surface [17–19]. The values of *σ*_*y*_ and *σ*_*z*_ are determined by the local atmospheric stability and the distance between the selected position and the pollutants. Atmospheric stability can be classified into six clusters (A-F) with the Pasquill-Gifford dispersion model [19, 20].

Plumes buoyantly rise to the height of Δ*H*, called the plume rise, before leveling off. According to Technical Guidelines for Environmental Impact Assessment: Atmospheric Environment in China (HJ/T2.2–93), the equation for plume rise is as follows:

When *Q*_*H*_ ≥ 2100*kW* and *T*_*s*_ − *T*_*a*_ ≥ 35*K*,

$$\Delta H=n_{0}Q_{H}^{n_{1}}H_{s}^{n_{2}}v^{-1}$$ (2)

$$Q_{H}=0.35P_{a}Q_{v}\frac{\Delta T}{T_{s}}$$ (3)

$$\Delta T=T_{s}-T_{a}$$ (4)

where *Q*_*H*_ is the heat release rate of the plume; the values of *n*_0_, *n*_1_, and *n*_2_ are selected by the technical guidelines; *P*_*a*_ is the atmospheric pressure (hPa); and *Q*_*v*_ is the actual smoke exhaust rate (m^3^/s). Thus, the equivalent height of the plume is *H* = *H*_*s*_ + Δ*H*, where *H*_*s*_ is the inherent height of the chimneys.

### Fuzzy matter-element analysis

#### Definition

Matter-element models are composed of objects, characteristics and values based on certain characteristics. If the values are fuzzy, the model is called a fuzzy matter-element model. The content and the relationship between the quality and the quantity of the comprehensive evaluation can be clearly illustrated. Fuzzy matter-element analysis has been widely used in many fields, including pattern recognition, scientific decisions and comprehensive evaluation [6]. The selection of atmospheric environmental monitoring sites is usually related to several PSIs; thus, the essence of the comprehensive evaluation of the atmospheric environment is a multiple attribute decision-making problem, and the optimized sites selected by every single indicator are usually incompatible. Fuzzy matter-element analysis is an effective method that can address such incompatibility problems; thus, it is used for the multiple attribute optimization problem of selecting sites for environmental monitoring. It is assumed that an ordered triple *R* = (*M*,*c*,*v*), including the three elements *M* (the matter), *c* (the matter’s property) and *v* (the property’s value), is defined as the basic cell, which is a dimensional matter element, and the matter is characterized with n properties (*c*_1_,*c*_2_, …*c*_*n*_). The corresponding values of these properties are (*v*_1_,*v*_2_, …*v*_*n*_). Then, a matter element is defined as follows [21–24]:

$$R=\left[\begin{array}{ccc} M & c_{1} & v_{1}\\ & c_{2} & v_{2}\\ & \vdots & \vdots \\ & c_{n} & v_{n} \end{array}\right]=\left[\begin{array}{c} R_{1}\\ R_{2}\\ \vdots \\ R_{n} \end{array}\right]$$

#### Establishment of matter element matrices

Matter element analysis models of each site were established to compare the PSIs of all sites, and each of the optimal values A (Min), the worst values B (Max) and the expected values C (Avg) was chosen. The expected value C constituted the two standard matter elements with the optimal value A and the worst value B. Their standard matter element matrices were given by

$$R_{AC}=\left[\begin{array}{ccc} M_{AC} & Q_{1} & \left(a_{1},c_{1}\right)\\ & \vdots & \vdots \\ & Q_{j} & \left(a_{j},c_{j}\right)\\ & \vdots & \vdots \\ & Q_{m} & \left(a_{m},c_{m}\right) \end{array}\right]\&R_{BC}=\left[\begin{array}{ccc} M_{BC} & Q_{1} & \left(b_{1},c_{1}\right)\\ & \vdots & \vdots \\ & Q_{j} & \left(b_{j},c_{j}\right)\\ & \vdots & \vdots \\ & Q_{m} & \left(b_{m},c_{m}\right) \end{array}\right]$$

The range of the joint domain matter elements constituted the optimal value A and the worst value B and became larger than the range of standard matter elements of each PSI. The joint domain matter element matrix was

$$R_{AB}=\left[\begin{array}{ccc} M_{AB} & Q_{1} & \left(a_{1},b_{1}\right)\\ & Q_{j} & \left(a_{j},b_{j}\right)\\ & Q_{m} & \left(a_{m},b_{m}\right) \end{array}\right]$$

Each site constituted measurements of all PSIs as a matter element:

$$R=\left[\begin{array}{ccc} M_{i} & Q_{1} & X_{i1}\\ & \vdots & \vdots \\ & Q_{j} & X_{ij}\\ & \vdots & \vdots \\ & Q_{m} & X_{im} \end{array}\right]$$

In these 4 matter element matrices, *a*_1_, *a*_2_, …*a*_*m*_ are the optimal values of each index; *b*_1_, *b*_2_, …*b*_*m*_ are the worst values of each index; *c*_1_, *c*_2_,…*c*_*m*_ are the expected values of each index; *Q*_1_, *Q*_2_ …*Q*_*j*_ are each PSI; *X*_*i*1_, *X*_*i*2_, …*X*_*im*_ are the measurements of Index *m* in Site *i*; *i* = 1, 2, 3 …*n*; and *j* = 1, 2, 3 …*m* [14, 21–24].

#### Solution to the comprehensive correlation function

To obtain representative monitoring indices, the optimized displayed average concentration of the PSIs should be consistent with that of the non-optimized concentration; that is to say, the measured indices should be close to the expected averages. Thus, the linear correlation functions of the PSI of each site for standard matter elements A and B were as follows:

$$K_{A}(x_{ij})=\frac{x_{ij}-c_{j}}{c_{j}-b_{j}}\&K_{B}(x_{ij})=\frac{x_{ij}-c_{j}}{c_{j}-a_{j}}$$

where *x*_*ij*_ is the measurement of Index *j* at Site *i*, *c*_*j*_ is the expected value of Index *j*, *a*_*j*_ is the optimal value of Index *j*, and *b*_*j*_ is the worst value of Index *j*.

If *K*_*A*_(*x*_*ij*_) ≥ 0, the compared object agrees with its requirement; the larger the value, the higher the degree of conformity. If −1 < *K*_*A*_(*x*_*ij*_) < 0, the compared object does not agree with its requirement, but it can be transformed into the standard object, and the larger the value is, the more easily the object can be transformed. If *K*_*A*_(*x*_*ij*_) < −1, the compared object does not agree with its requirement nor can it be transformed into the standard object [14]. Therefore, the comprehensive correlation function of all PSIs for A and B can be calculated as follows:

$$K_{A}(x_{j})=\sum_{j=1}^{m}W_{j}\times K_{A}(x_{ij})\;\&\;K_{B}(x_{j})=\sum_{j=1}^{m}W_{j}\times K_{B}(x_{ij})$$

where *W*_*j*_ is the normalization weight, which can be calculated by the index exceeding method [14, 24] of atmospheric environmental quality grading.

### Materials

The Pinghu refuse incineration power plant in Shenzhen, Guangdong Province, China, was chosen as an example. The latitude and longitude of this plant are 114.101621°E and 22.680891°N, respectively. The single-day processing capacity of refuse at this plant is 1675 tons. The height of the smoke vent is approximately 80 meters. At the beginning of production, due to misused funds and mismanagement, the pollutant emissions exceeded standards at one time and led to residents protesting. The technical standard in China stipulates that the monitoring range for a high-elevation point source can extend to 500–4000 meters. Thus, the survey region was set as a radius of 5 kilometers around the center of the plant. The wind velocity and direction data for nearly three years were provided by the Shenzhen Meteorological Bureau. The frequency of wind direction by season and the average wind velocity were calculated. The parameters from the 3^rd^ season were used as the objects of the study. The source intensity (g/s) and outlet temperature of the pollution source can be calculated from the chimney’s height, diameter, velocity of flue gas, elements in the refuse, and other parameters. To simplify the model, only the limited values of total suspended particulates (TSP), SO_2_ and NOx were examined in the study.

#### Calculation of predominant wind direction

**Fig 1:**
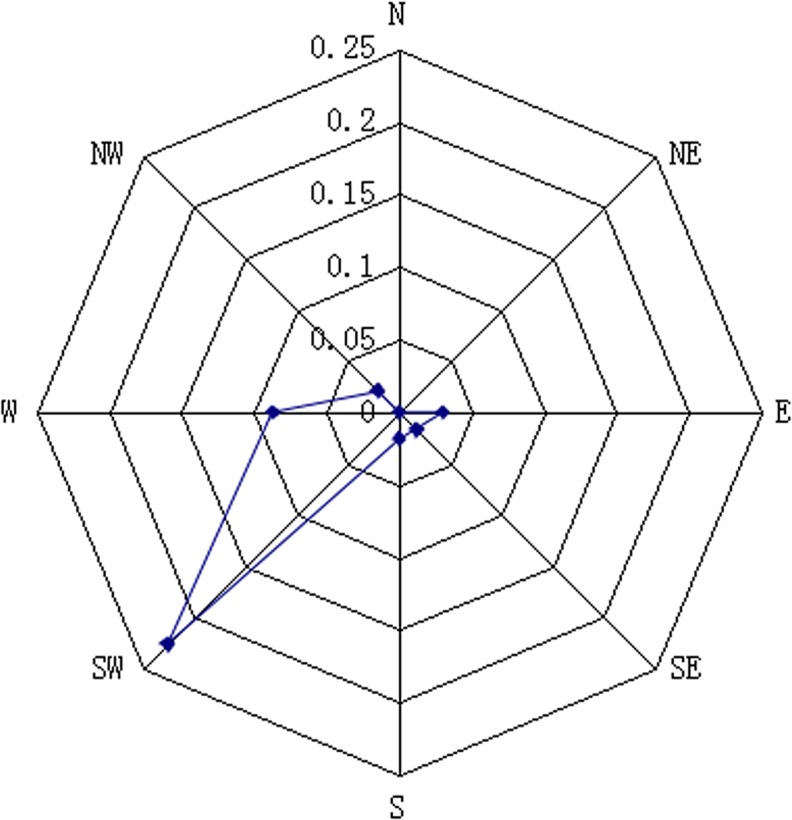
The distribution of the contamination factor. According to the meteorological data, southwesterly was the predominant wind direction in the 3^rd^ season.

According to Gaussian atmospheric diffusion, the visible contouring of a plume is generally distributed in the range of 45° downwind. For simplicity, the wind directions were divided into eight groups—E, SE, S, SW, W, NW, N and NE—so that these eight directions covered 360°. According to the Gaussian plume model, the concentration of the plume is proportional to the source intensity of the pollution source and inversely proportional to wind velocity; thus, a “contamination factor” is defined as wind frequency/average wind velocity. The higher the factor of a certain direction is, the higher the probability that the direction is polluted. Thus, to monitor cost savings, the monitoring sites were only located in the wind direction whose “contamination factor” was high. Using the meteorological data, the wind rose of the contamination factor in the 3^rd^ season was drawn, as shown in Fig 1. Southwesterly was the predominant wind direction.

#### Extraction of geographic parameters by GIS

The latitude, longitude and elevation of each site were automatically and equidistantly extracted and numbered using ArcGIS software. In this case, sites were established every 60 meters for a total of 2705 sites.

#### Transformation of geographic parameter coordinates into analytical geometry coordinates

The longitude and latitude coordinates were transformed into rectangular coordinates using ArcMap so that the pollution source is the origin, axis *Y* points due north and axis *X* points due east.

#### Rotation of coordinates

Because one of the preconditions of the revised Gaussian mirror-image plume equation is that the wind direction must be the positive direction of axis *X*, the entire rectangular coordinate system *XOY*, which is centered on the origin, should rotate for different wind directions in the horizontal. When there are several predominant wind directions in the same season, ArcMap is used to select equidistant sites and extract geographic parameters in the area covered by each predominant wind direction, and these coordinates are then rotated. Finally, the monitoring sites for each predominant wind direction are confirmed.

#### Calculation of the pollutant concentration of each site

All of the parameters in Eqs 1, 2, 3 and 4 reflect the actual conditions. Eq 2 and the geometric parameters were input into MATLAB. The concentration of each pollutant at all sites in the predominant wind direction could then be calculated. Because there is a certain background concentration of the pollutants in nature, when the pollutant concentration of each site was calculated, the background concentration was added. The background concentration is different between urban and rural areas. In this case, because the area downwind is mainly urban, the background concentration of all sites was considered the same as that of the urban area. The data for urban background concentration were provided by the Shenzhen Environmental Protection Bureau. According to Annual State of Environment in Shenzhen (S1 Text), the background concentrations of TSP, SO_2_ and NOx were 0.062, 0.011 and 0.04 mg/m^3^, respectively.

#### Using fuzzy matter-element analysis to define the coordinates of monitoring sites

Step 1 Statistical analysis of A, B and C of the three types of pollutants in the predominant wind direction (Table 1) was conducted, and the standard matter element matrices were established for A, B and C. The sites were filtered for the first time, and the initial sites at which the relative error between the predicted values and the expected values was less than 2% were confirmed.

**Table 1:** The components of matter element matrices.

Wind direction	Pollutants	Max *b*_*j*_ (mg/m^3^)	Min *a*_*j*_ (mg/m^3^)	Avg *c*_*j*_ (mg/m^3^)
Southwester	TSP	0.58670659	0.062	0.123260345
	SO_2_	0.873416682	0.011	0.111688547
	NOx	1.164769977	0.04	0.171318719

The matter element matrices of pollutants in the southwest are made up of the maximum, minimum and average concentrations of the three kinds of pollutants.

Step 2 Comprehensive correlation functions *K*_*A*_(*x*_*j*_) and *K*_*B*_(*x*_*j*_) for each site were calculated. The index-exceeding method based on Ambient Air Quality Standards in China (GB3095-2012) was used to calculate the normalization weight *W*_*j*_ [21–24]. Eq 2 and *W*_*j*_ were used to calculate the linear correlation functions *K*_*A*_(*x*_*j*_) and *K*_*B*_(*x*_*j*_), which are shown in Table 2.

**Table 2:** Comprehensive correlation functions.

Site number	*K*_*A*_(*x*_*j*_)	*K*_*B*_(*x*_*j*_)	Site number	*K*_*A*_(*x*_*j*_)	*K*_*B*_(*x*_*j*_)
**114**	-0.001922056	0.014540719	**1889**	0.000019814	-0.000149894
**1761**	-0.001829489	0.013840438	**2298**	0.000165748	-0.001253917
**1932**	-0.001771437	0.013401262	**1588**	0.00032467	-0.002456191
**2299**	-0.001662829	0.012579621	**1769**	0.000567869	-0.004296036
**1915**	-0.001520314	0.01150147	**1644**	0.000619253	-0.004684768
**1711**	-0.001511911	0.011437897	**1750**	0.000701402	-0.005306241
**839**	-0.001290948	0.009766265	**1740**	0.000759464	-0.005745489
**2303**	-0.001164124	0.008806822	**1203**	0.000789228	-0.005970658
**1616**	-0.000773325	0.005850348	**1701**	0.000840024	-0.006354943
**1663**	-0.000537436	0.004065804	**2304**	0.001068236	-0.008081407
**1682**	-0.000400278	0.003028181			

The comprehensive correlation functions *K*_*A*_(*x*_*j*_) and *K*_*B*_(*x*_*j*_) were calculated, and these two parameters would make up the scatter diagram of the sites, which can be selected as the final monitoring sites.

**Fig 2:**
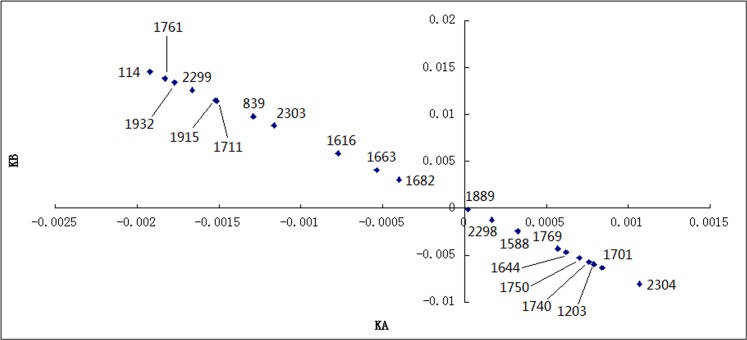
The scatter diagram of the sites. The scatter diagram of the sites was drawn with *K*_*A*_(*x*_*j*_) as axis *X* and *K*_*B*_(*x*_*j*_) as axis *Y*, and Site 114, 1761, 1932, 1889 and 2304 were selected as the monitoring sites.

Step 3 The scatter diagram of the sites can be drawn with *K*_*A*_(*x*_*j*_) as axis *X* and *K*_*B*_(*x*_*j*_) as axis *Y* (Fig 2). According to matter element theory [17], when the point is located in Quadrant IV, the greater the distance between the points and the coordinate axis, the more accurately *K*_*A*_(*x*_*j*_) is represented; when the point is located in Quadrant IV, the greater the distance between the points and the coordinate axis, the more accurately the *K*_*B*_(*x*_*j*_) is represented; when the point is located near the origin, it indicates that the shorter the distance between the points and the origin, the more easily the points can be transformed into the optimal values or the worst values. To ensure that the data measured by the monitoring sites synthetically represent the local pollution level, sites from each of these three types were taken as the candidate monitoring sites.

Step 4 The calculated coordinates were transformed inversely into latitude and longitude.

## Results

**Fig 3:**
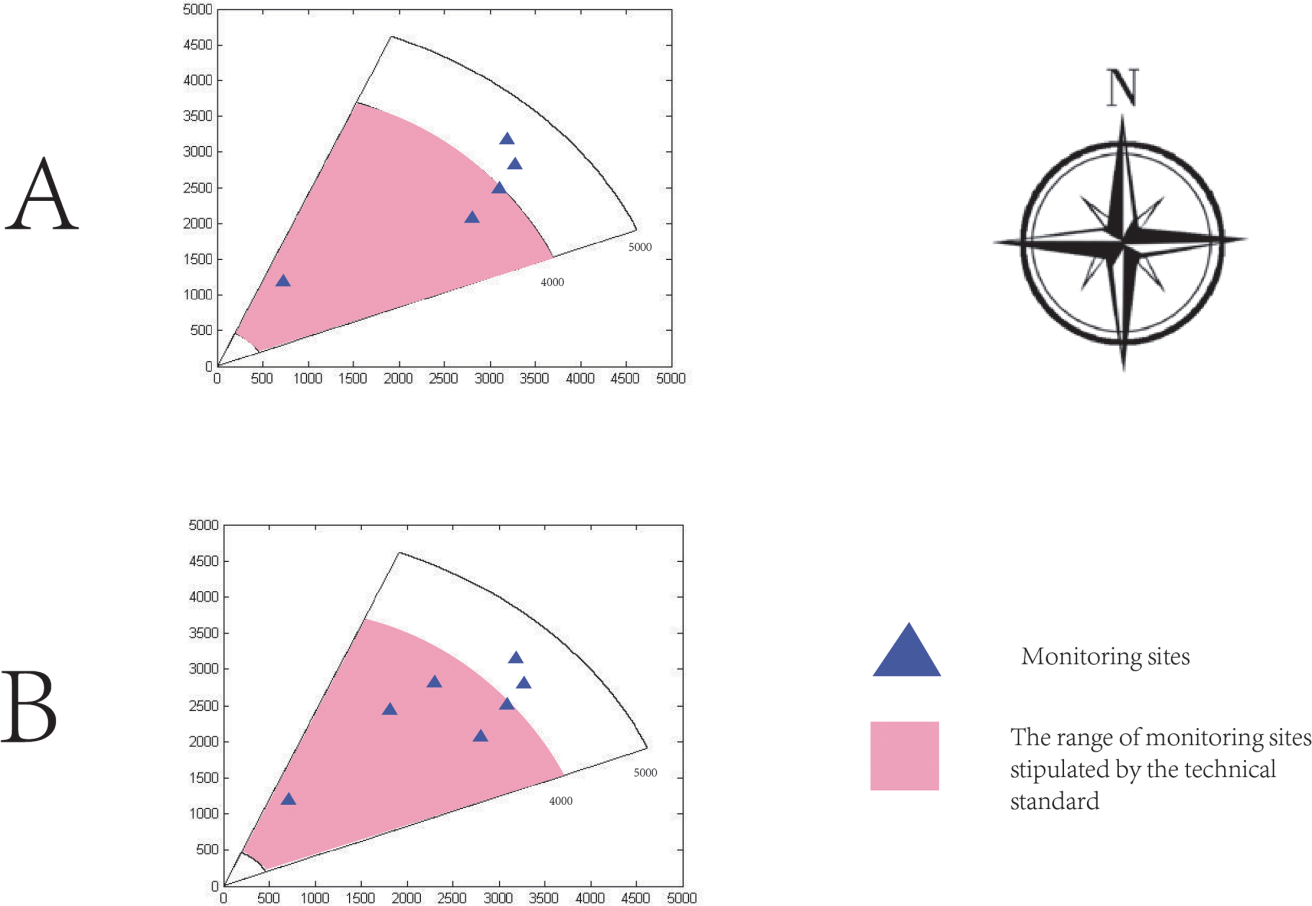
The positions of monitoring sites. (A) The actual positions of monitoring sites determined using fuzzy matter-element analysis. (B) The actual positions of monitoring sites that were improved using the hierarchical clustering method. The methods shown in (A) and (B) provide similar results.

As shown in Fig 2, the distance between the coordinate axis and Site 2304 was the greatest. Site 2304 most accurately represents *K*_*A*_(*x*_*j*_) (the optimal values); therefore, it should be selected as a monitoring site. The distances between the coordinate axis and Sites 114, 1761 and 1932 were the greatest. These sites most accurately represent *K*_*B*_(*x*_*j*_) (the worst values); therefore, they should be selected as monitoring sites. Near the origin, the distance between Site 1889 and the origin was the shortest, indicating that the data from this site could be most easily transformed into either the optimal value or the worst value. Thus, this site should also be selected as a monitoring site. The analytical geometry coordinates, latitude and longitude of all the selected monitoring sites are shown in Table 3, and the specific locations are shown in Fig 3A. The technical standard stipulates that the range of monitoring sites can expand to a radius of 500–4000 meters in consideration of the impact of high-elevation point sources on concentrations on the ground. As shown in Table 3, the distance between the sites selected and pollution sources is generally in accord with the national standard; thus, the selection is reasonable.

**Table 3:** The coordinates of atmospheric environmental monitoring sites in the 3^rd^ season.

Site number	*X*	*Y*	*Z*	Latitude	Longitude
**114**	673.607	1227.906	61.474	114.104735	22.696915
**1761**	3127.677	3190.019	81.709	114.129279	22.713627
**1932**	3034.455	2526.048	49.542	114.12811	22.707698
**1889**	2750.525	2094.106	52.583	114.125188	22.703924
**2304**	3202.903	2833.08	45.859	114.129864	22.710393

*X*, *Y* and *Z* are the analytical geometry coordinates, *Z* is elevation. These sites were finally selected according to the positions of the sites in the scatter diagram.

**Fig 4:**
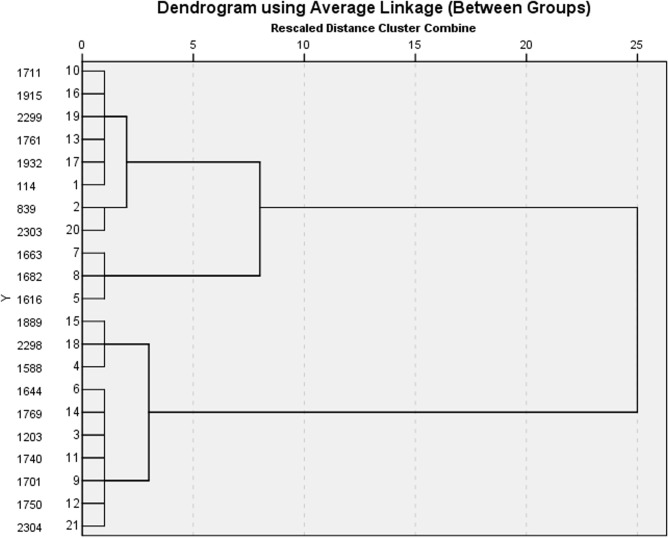
The dendrogram of the hierarchical clustering method for monitoring sites. The hierarchical clustering method was used to select the monitoring sites, and besides the five same sites shown in Table 3, another two sites: 1616 and 839 were selected.

To test the rationality of this method, the hierarchical clustering method in SPSS 19 was used to analyze the data in Table 1 [25, 26]. The relationship among the sites was evaluated based on Euclidean distance of the PSI at each site using the between-groups linkage method. A cluster dendrogram was drawn (Fig 4), and the sites could be classified into five types: Site 114, 1761 and 1932 were the same type; Site 1889 was a single type; and Site 2304 was a single type. Two other types represented by a single site were Sites 1616 and 839, whose latitude and longitude are shown in Table 4. The actual locations of these five types of sites are shown in Fig 3B. Compared with Fig 3A, in Fig 3B, only two alternative positions of monitoring sites were added by the hierarchical clustering method, and the locations of other sites did not change. Therefore, there is no radical change between the hierarchical clustering method and fuzzy matter-element analysis. In fact, when the coordinates of the sites selected by the two methods were input into GIS, the classifications according to these two methods were similar (Fig 4). In these five types of sites classified by the hierarchical clustering method, the actual locations of sites within the same type were adjacent, such as for Sites 1663 and 1682. Thus, in the actual selection of monitoring sites, it is proposed that fuzzy matter-element analysis and hierarchical clustering method should be comprehensively combined with actual conditions to select the sites. The monitoring agency can establish the mobile platforms to constantly ensure that the range of moving and sampling are within the sites selected by these two methods.

**Table 4:** The coordinates of complementary sites.

Site number	*X*	*Y*	*Z*	Latitude	Longitude
**1616**	2236.307	2852.928	55.614	114.120514	22.710931
**839**	1769.128	2473.432	63.452	114.115839	22.707696

These two sites were selected by the hierarchical clustering method. Besides these two sites, another five sites selected by the hierarchical clustering method were same as the sites selected by fuzzy matter analysis.

## Discussion

Previous works on the selection of atmospheric environmental monitoring sites have focused on the application of mathematics and have emphasized field measurements [27–30]. Silva C et al. optimized Santiago’s atmospheric monitoring networks by using Shannon information index, and achieved the low cost modifications of the networks [27]; Mazzeo NA et al. designed a multiple objective and multi-pollutant planning procedure based on the statistical analysis, the DAUMOD and the AERMOD atmospheric dispersion models to monitor the air quality in the city of Buenos Aires [28]; Pope R et al. developed a GIS-based, multi-objective assessment approach that integrated environmental, economic, and social indicators to assess monitoring networks in the Phoenix metropolitan area [29]; Gómez-Losada Á et al. devised a combination of formal statistical methods, which combined the expectation maximization (EM) algorithm, the hierarchical clustering analysis (HCA), the principal component analysis (PCA) and such statistical methods closely to monitor the air quality in Andalusia, Spain [30]. Though these approaches adopted the rigorous mathematic methods to show the rationality of their optimizing processes, the final optimized sites are still chosen from the existing sites, which are restricted by the historical factors, the geographical conditions and so on. However, these studies did not explain the rationality of these existing sites. On the other hand, these previous studies are mostly suitable for monitoring a large area such as a city, instead of a point pollutant source like a single factory or power plant, so all of them are not proposed to the method to optimize the monitoring sites of the point pollutant source effectively. On the contrary, this study focuses on monitoring the small-scale pollutant source, which not only uses rigorous mathematic methods to optimize the positions of the sites but also concentrates on researching and exploring the functions of GIS in environmental monitoring. The positions of the sites are rebuilt by this study, rather than influenced by the existing monitoring sites, which avoid discussing the rationality of the initial sites. This study combines mathematic methods and computer simulation so that monitoring sites can be predicted without field measurements. Such, the cost of monitoring can be decreased.

This method has shown its merits in the study. It is easy to combine geographic parameter extraction by GIS and fuzzy matter-element analysis. This method can be directly implemented by programming. The results of the assessment are intuitive and accurate and similar to the results found by the hierarchical clustering method. The cost is much lower than the mesh point method. The method is highly practicable, and it can be used in monitoring the impact of an isolated high-elevation point source, such as a single factory, on the local area. However, using fuzzy matter-element analysis alone can only optimize the pre-existing monitoring sites; thus, this method is used to monitor a large area such as a city rather than a localized area. However, this method also has some defects. Both fuzzy matter-element analysis and the hierarchical clustering method are based on calculations to classify the sites (the former is based on the comprehensive correlation function, and the latter is based on the Euclidean distance of the PSI at each site). These methods cannot reflect the spatial relationship and the on-the-ground configuration of the sites. The actual topographic conditions should be considered comprehensively to determine the final optimization solution. Of course, to ensure the accuracy of the selection, on-site investigations are suitable, but the number of potential sites can be decreased by this type of the study; that is say, the number of on-site investigations can be decreased, and the cost of monitoring can be greatly reduced.

In this case, for ease of calculation, the quantity of pollutants was assumed to be constant, and dispersion was neglected. Meanwhile, because the whole area covered by the predominant wind direction was urban, the background concentration was not distinguishable. These factors will influence the accuracy of the results to some extent. In addition, more comparisons will be helpful to test the performance of this method, such as making a comparison of the measured results with the predictive values at the selected sites, or comparing the performance of optimizing the monitoring sites among the different methods. These factors and comparisons would be analyzed further.

## Supporting Information

S1 Dataset Supporting Information file. All the information of the monitoring sites in the study is available as Supporting Information files, including the sites number, Mercator coordinates, altitude, the rectangular coordinates transformed by Mercator coordinates (*X*, *Y*, *Z*), the rectangular coordinates after rotating the coordinates axis (*X*, *Y*, *Z*), latitude (E), longitude (N) and the concentration of TSP, SO_2_ and NOx (mg/m^3^).
(XLS)

S1 Text Supporting Information. The meteorological data of the study area can be searched in the website of Shenzhen Meteorological Bureau: http://www.szmb.gov.cn/. E-mail: webmaster@szmb.gov.cn. The pollutant background concentration of the study area can be searched in the web site of Shenzhen Environmental Protection Bureau: http://www.szepb.gov.cn/.
(DOC)
