# Supplementary material for: Selection of Atmospheric Environmental Monitoring Sites based on Geographic Parameters Extraction of GIS and Fuzzy Matter-Element Analysis
Source: PLoS One. 2015 Apr 29;10(4):e0123766. doi: 10.1371/journal.pone.0123766 (PMC4414522; doi:10.1371/journal.pone.0123766)
Supplement: S1 Text — The meteorological data of the study area can be searched in the website of Shenzhen Meteorological Bureau: http://www.szmb.gov.cn/. E-mail: webmaster@szmb.gov.cn. The pollutant background concentration of the study area can be searched in the web site of Shenzhen Environmental Protection Bureau: http://www.szepb.gov.cn/. (DOC) [file pone.0123766.s002.doc]

**S1_Text.** All the information of the monitoring sites in the study is available as Supporting Information files, including the sites number, Mercator coordinates, altitude, the rectangular coordinates transformed by Mercator coordinates (*X*, *Y*, *Z*), the rectangular coordinates after rotating the coordinates axis (*X*, *Y*, *Z*), latitude (E), longitude (N) and the concentration of TSP, SO_2_ and NOx (mg/m^3^). The meteorological data of the study area can be searched in web site of Shenzhen Meteorological Bureau: <http://www.szmb.gov.cn/>. E-mail: [webmaster@szmb.gov.cn](mailto:webmaster@szmb.gov.cn). The pollutant background concentration of the study area can be searched in the web site of Shenzhen Environmental Protection Bureau: <http://www.szepb.gov.cn/>.
